# Attentional set to safety recruits the ventral medial prefrontal cortex

**DOI:** 10.1038/s41598-018-33953-3

**Published:** 2018-10-18

**Authors:** Shuxia Yao, Song Qi, Keith M. Kendrick, Dean Mobbs

**Affiliations:** 1 The Clinical Hospital of Chengdu Brain Science Institute, MOE Key Laboratory for NeuroInformation, University of Electronic Science and Technology of China, Chengdu, Sichuan 611731 China; 2 California Institute of Technology, Pasadena, California 91125 USA; 3 Columbia University in the City of New York, New York, NY 10027 USA

## Abstract

Early detection of danger is highly adaptive, yet fast orientation towards safety is also key to survival. This study aimed to explore how human brain searches for safety by manipulating subjects’ attentional set. Subjects were asked to judge random dots motion (RDM) direction and could be shocked for incorrect responses (RDM trials) while keeping alert in detecting shock probability cues (cue detection trials). Relative to safe condition, where attention was set to search cues associated with no shock, incorrect responses to ‘dangerous+’ cues would increase and correct responses to ‘dangerous−’ cues would decrease shock probability. In RDM trials, relative to the ‘dangerous+’, the safe and ‘dangerous−’ attentional set induced stronger activation in the ventral medial prefrontal cortex (vmPFC), a core region involved in flexible threat assessment and safety signalling. In cue detection trials, shorter response times and greater accuracy were observed for ‘dangerous+’ than ‘dangerous−’ and safe cues. At neural level ‘dangerous+’ cues induced stronger activity in the frontoparietal attention network than safe cues. Overall, our findings demonstrate that attentional set for searching safety recruits the vmPFC, while detection of threat-related cues elicits activity in the frontoparietal attention network, suggesting new roles for these regions in human defensive survival circuitry.

## Introduction

Our attention systems have evolved to detect stimuli that are of survival value, with early detection of potential ecological dangers being of crucial importance. During threat assessment, the detection of threat *per se* is only one among several other parallel strategies including threat monitoring, prediction and safety seeking^1^. In the case of searching for safety, the organism will search the environment for a safe refuge and in turn, this will influence its decision to either freeze or flee. For example, it has been theorized that when escape is viable flight will occur, but when it is not then freezing will be the choice of defense^2^. Consequently, decreased fear induced by the knowledge of being safe facilitates exploration of the environment by organisms and thus increases their foraging and copulation opportunities^3,4^. How the human brain implements this safety search strategy is unknown.

Animal studies have shown that access to safety cues can affect survival responses. More specifically, it has been revealed that safety cues can abolish analgesia, a typical defensive response elicited by threat^5^. Fear conditioning studies in animals have further demonstrated that learned safety (in the context of the unpaired neutral vs. the aversive conditioned stimulus) is associated mainly with the medial prefrontal cortex (mPFC) and basolateral amygdala^6,7^. However, there are very few human studies which have investigated mechanisms underlying safety searching, with initial evidence showing that access to safety can decrease fear responses either in healthy populations^8,9^ or in patients with affective disorders such as panic disorder and claustrophobia^10,11^. Given that safety searching is closely associated with defensive responses^2^, the defensive survival system may therefore be involved in safety processing.

Threat can evoke different defensive behaviors depending on the distance between the predator and the prey, as proposed by Fanselow and Lester (1988) in their “Threat Imminence Continuum” model^12^. Two main defensive survival systems have been recognized in animal studies. One is the ‘cognitive fear’ circuitry in processing distal threat, consisting of corticolimbic regions such as medial prefrontal cortex (mPFC), anterior cingulate cortex (ACC), amygdala, and hippocampus, allowing elaborate information processing and promotion of behavioral flexibility^1,13–16^. When threat becomes imminent, the ‘reactive fear’ circuitry, encompassing the midbrain and hypothalamus, will be evoked to initiate fast reactions such as freezing or fleeing^14,17,18^. These two circuits switch between each other according to the spatial distance to threat and thus can optimize defensive responses. Human fMRI studies have demonstrated a highly similar survival system in humans to that found in animal models. In this system, as predators move from a distal to close distance, the regions involved switch from the forebrain, including the vmPFC, rostral ACC (rACC), amygdala and hippocampus, to the midbrain periaqueductal gray^19–21^. Because elaborate evaluation of the environment, including safety searching, can only occur when threat is distal or absent, the ‘cognitive fear’ circuitry may thus be more closely implicated in safety searching. This hypothesis can be supported by findings that the vmPFC, which is also a core hub of the ‘cognitive fear’ circuitry, is involved in both the learned safety and safety signaling^21–26^.

**Figure 1:**
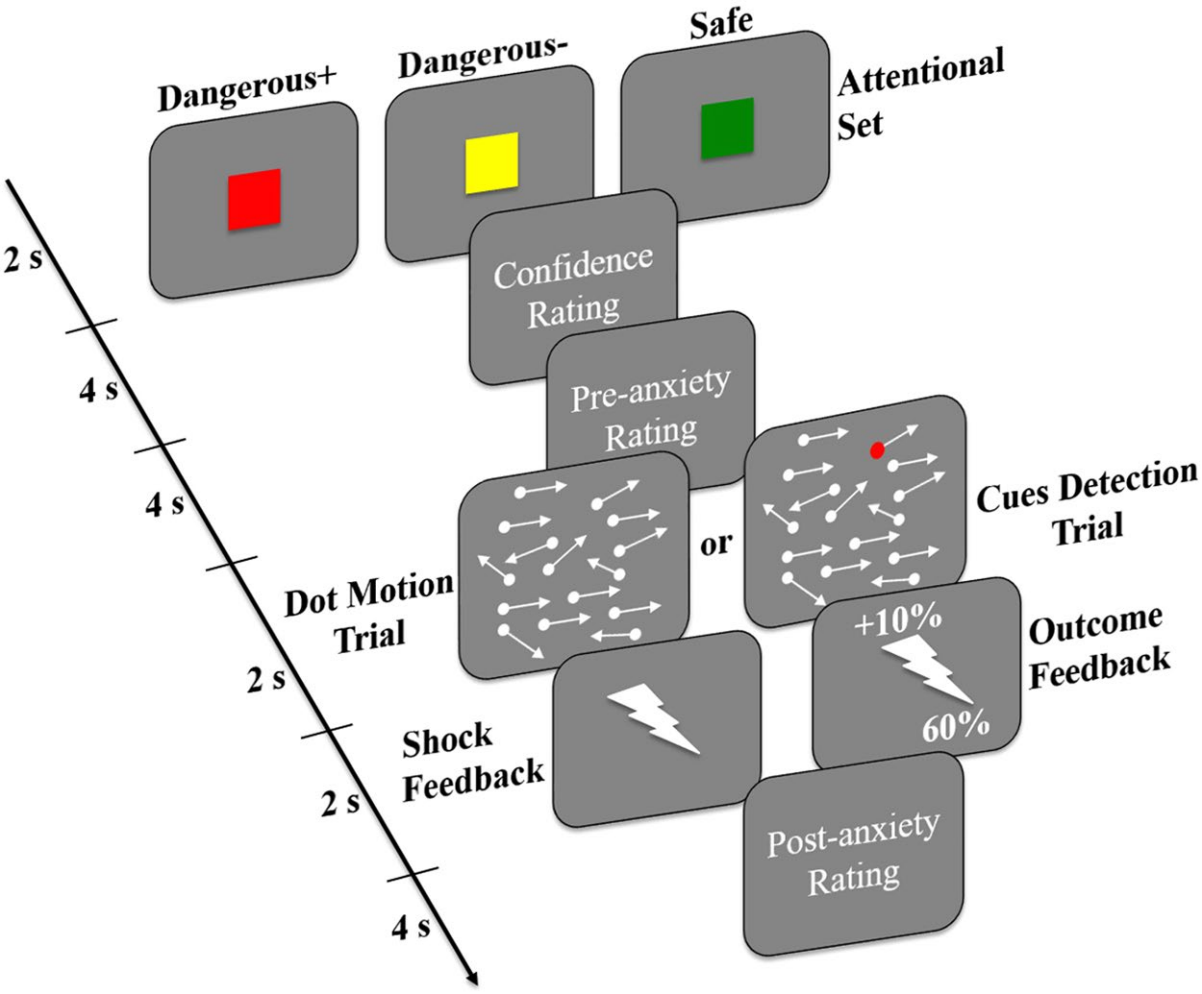
Experimental task. In random dots motion discrimination trials, subjects were asked to judge the moving direction by pressing the ‘left’ or ‘right’ buttons. For cues detection trials, subjects were informed that the colored dot would appear at any time point during the 2-s white dots screen and were instructed to respond as fast as possible before it disappeared. Note that arrows indicate the moving direction of the dots rather than the stimuli *per se* and that the ‘dangerous+’ condition was used as an example in the figure.

The present study investigates the neural mechanism underlying safety searching by manipulating attentional set to safe or dangerous cues using a novel dot-motion paradigm combined with electric shocks (Fig. 1). In this paradigm, subjects were asked to judge dot-motion direction while simultaneously keeping alert to the emergence of safety or danger cues that were associated with either a neutral or aversive outcome (electric shocks). Subjects could be shocked for incorrect responses in the dangerous condition and the shock probability depended on subjects’ performance. Given that previous studies have highlighted a specific role of the vmPFC in both the learned safety and safety signaling^21–26^, we predicted that the vmPFC would be a core region involved in attentional set to safety search. We additionally predicted that the goal directed attentional network could be involved in searching for dangerous cues^27–29^, driven by the higher motivation level occurring in response to cues signaling danger relative to safety^30,31^.

## Results

### Behavioral results

#### Confidence and anxiety ratings

**Figure 2:**
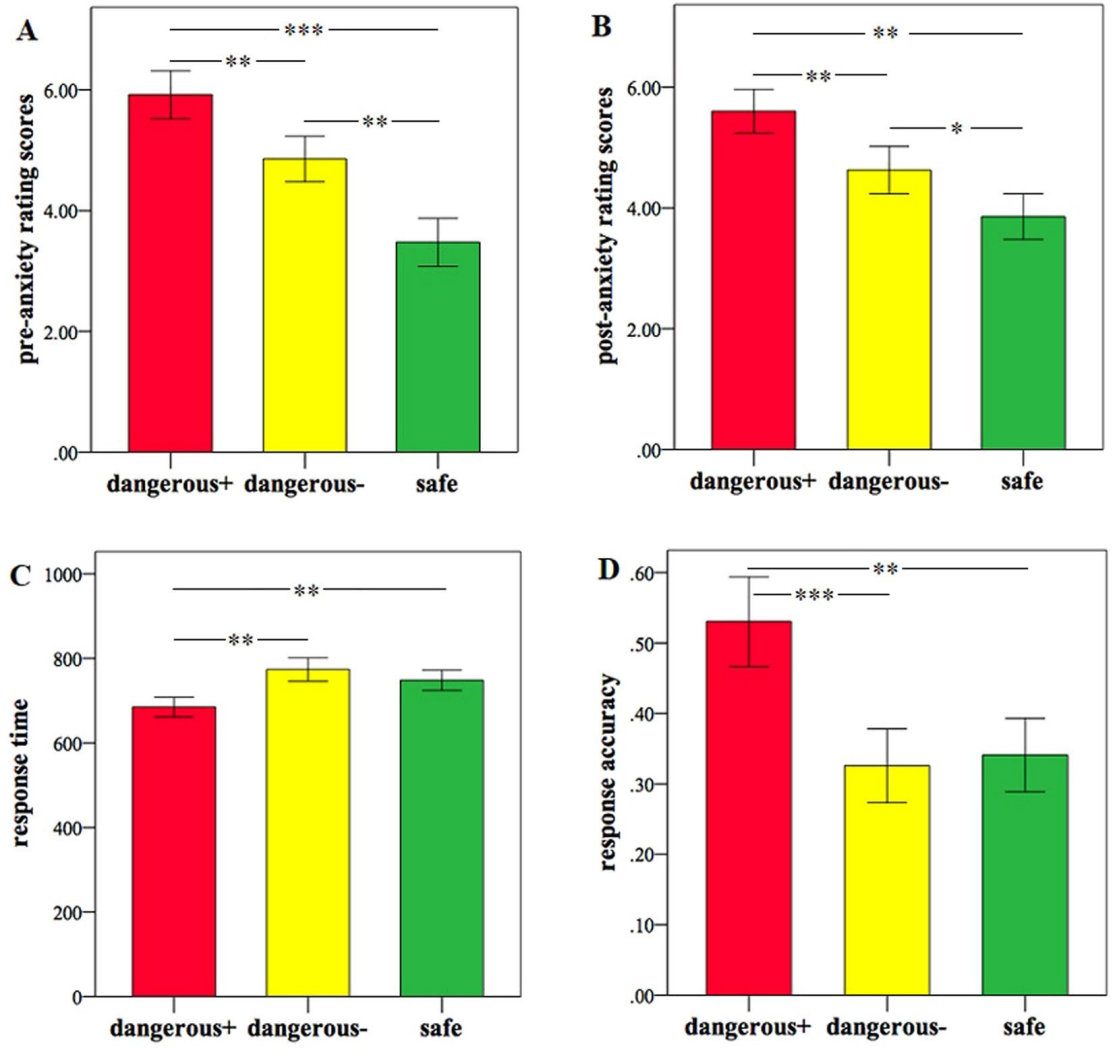
(**A**) Mean pre-anxiety rating scores before each block in each threat condition. (**B**) Mean post-anxiety rating scores after each block in each threat condition. Mean response time (**C**) and accuracy (**D**) to each cue condition in cue detection trials. *P < 0.05; **P < 0.01; ***P < 0.001.

For rating scores, one more subject had to be excluded due to failure of rating data acquisition (see Methods). A repeated-measures ANOVA on the confidence rating scores with threat condition (D+ vs. D− vs. safe) as a within-subject factor revealed a significant main effect (F(2, 40) = 5.91, p = 0.017), with a trend of decreased confidence in the D+ (p = 0.052; mean ± SD = 4.94 ± 1.72) and D− (p = 0.074; 5.49 ± 1.45) conditions relative to the safe condition (6.00 ± 1.48). For the pre-anxiety ratings, there was a significant main effect of threat condition (F(2, 40) = 20.08, p < 0.001). Post-hoc tests showed that subjects were more anxious in performing the D+ than the D− (p = 0.003) and safe conditions (p < 0.001) and were more anxious in performing the D− than the safe conditions (p = 0.004) (Fig. 2A). A significant main effect was also found for the post-anxiety ratings (F(2, 40) = 15.33, p < 0.001), with subjects more anxious in the D+ than the D− (p = 0.007) and safe conditions (p = 0.001) and more anxious in the D− than safe conditions (p = 0.010) (Fig. 2B).

#### RDM trials

To test the difference of attentional set between different threat conditions and its potential interaction effects with difficulty on the behavioral level, we performed a repeated-measures ANOVA on RT and RA with threat condition (D+ vs. D− vs. safe) and difficulty levels (difficult vs. moderate vs. easy) as within-subject factors. For RT, this analysis revealed a significant main effect of difficulty (F(2, 42) = 77.40, p < 0.001). Post-hoc analysis showed that, as expected, subjects responded faster in the easy than in the difficult (p < 0.001) and moderate levels (p < 0.001) and faster in the moderate than in the diffcult levels (p = 0.009) (Fig. S1A). For RA, there was a significant main effect of difficulty (F(2, 42) = 108.08, p < 0.001), with subjects exhibiting a higher accuracy in the easy than in the difficult (p < 0.001) and moderate levels (p < 0.001) (Fig. S1B). There were no other significant main effects or interactions (ps > 0.067).

#### Cue detection trials

To test the difference of behavioral performance in searching for shock proability cues signalling danger or safety, a repeated-measures ANOVA on RT with threat as within-subject factor revealed a significant main effect of threat (F(2, 42) = 11.93, p < 0.001), with faster responses to the D+ cue compared to the D− (p = 0.001) and safe cues (p = 0.003; Fig. 2C). For RA, the main effect of threat was also significant (F(2, 42) = 10.78, p < 0.001). Post-hoc tests showed that the RA was higher in the D+ cue relative to the D− (p < 0.001) and safe cues (p = 0.007; Fig. 2D).

### fMRI results

#### RDM trials

**Figure 3:**
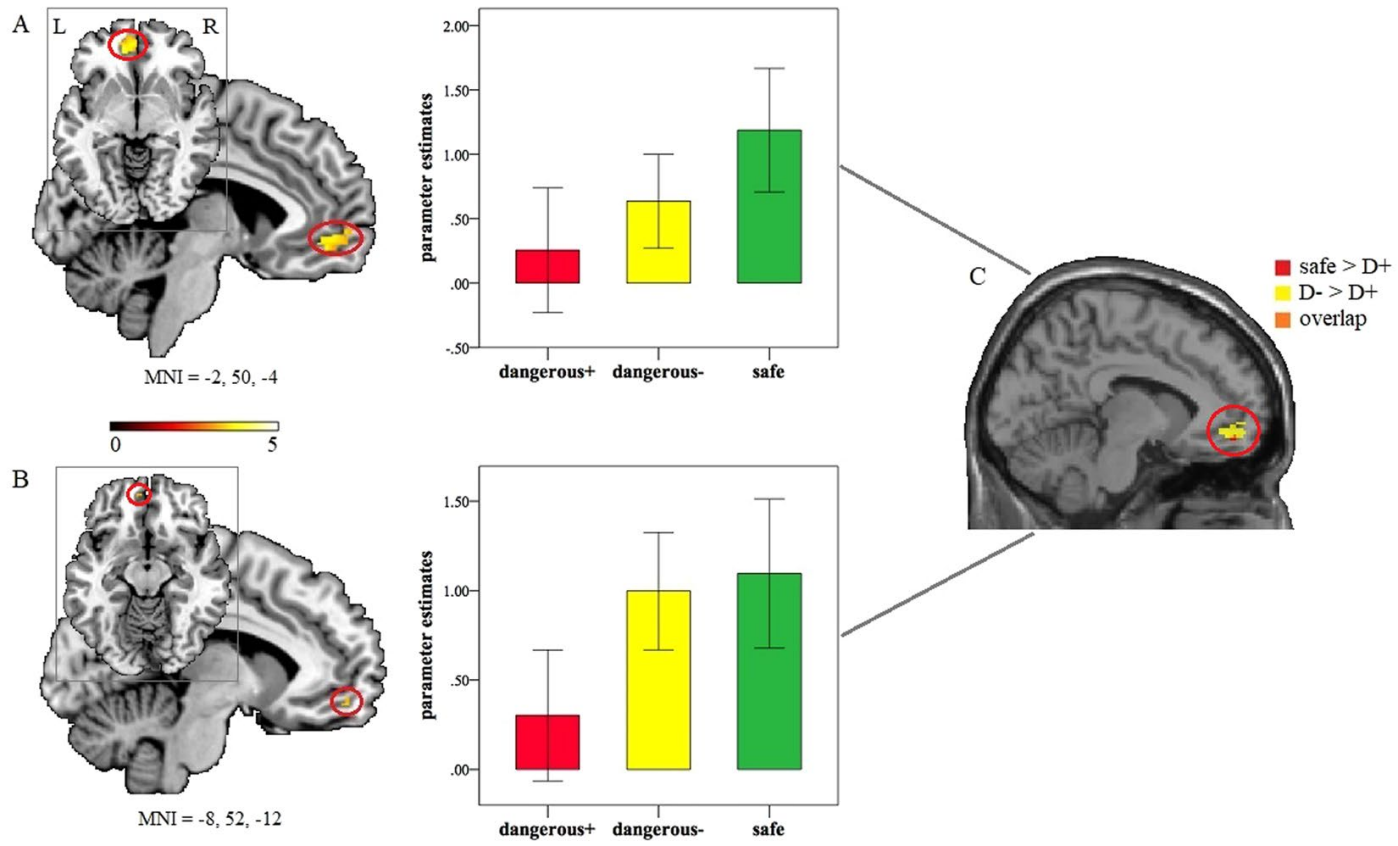
Increased left vmPFC activity in response to (**A**) the safe relative to D+ threat conditions (safe > D+) and (**B**) to the D− relative to D+ threat conditions (D− > D+) in random dots motion trials. (**C**) Overlap between the ‘safe > D+’ and ‘D− > D+’ comparisons. Parameter estimates presented in bar graphs were extracted from a 6-mm sphere centered on the peak coordinates. Statistic maps were displayed with a P < 0.001 uncorrected threshold. L: left. R: right. D+: ‘dangerous+’. D−: ‘dangerous−’.

To specifically identify the neural effects associated with attentoinal set in different threat conditions, particlularly attentional set to safety that was our primary interest, we first conducted a hypothesis-driven ROI analysis between threat conditions. Comparisons between safe and D+ conditions revealed stronger activation in the bilateral vmPFC (left: MNI = −2, 50, −4; t = 4.54; P_FWE_ = 0.001; voxels = 126; Fig. 3A; right: MNI = 4, 48, −12; t = 3.97; P_FWE_ = 0.006; voxels = 77) for attentional set to safety relative to the D+ cues (safe > D+). Furthermore, increased activity was observed in the left vmPFC (MNI = −8, 52, −12; t = 3.46; P_FWE_ = 0.024; voxels = 4; Fig. 3B) when attention was set to D− compared to the D+ cues (D− > D+). Given the left vmPFC only has 4 voxels, we further confirmed this effect by extracting the parameter estimates using an independent coordinate (MNI = −6, 51, −15) associated with safety processing in a previous study^23^. A paired t-test revealed a significantly increased activity of the left vmPFC in the D− (mean = 1.18, SD = 1.79) compared with the D+ (mean = 0.29, SD = 1.76) conditions (t(21) = 2.91, P = 0.008). Importantly, the left vmPFC overlapped between the ‘safe > D+’ and ‘D− > D+’ comparisons (Fig. 3C). To exclude the possibilty that the different number of delivered shocks in D+ (mean = 6.59, SD = 3.57) and D− conditions(mean = 4.59, SD = 2.58) may confound the findings, we examined whether shock number was associated with the vmPFC activity but found no significant correlations (ps > 0.368). No signifincat effects were found for comparisons between safe and D− conditions and for other comparisons associated with threat conditions (e.g., D+> safe or D− > safe) in the a priori ROIs (P_FWE_ < 0.05). For the main effect of difficulty levels and interactions between threat condition and difficulty levels, no significant effects were observed in the a priori ROIs (P_FWE_ < 0.05).

In the whole brain analysis, we only observed significantly increased activity in left rACC, left dmPFC, right vmPFC, right hippocampus and bilateral insula in the safe relative to the ‘dangerous+’ conditions (safe > D+; P_FDR_ < 0.05) (Table 1). Comparisons between difficulty levels showed stronger activation in bilateral inferior frontal gyrus, insula, dmPFC and lingual gyrus and other regions for more difficult than easier tasks (see Table S1). However, there were no other significant main effects and interactions between threat condition and difficulty levels in regions out of the a priori ROIs at the whole brain level (P_FDR_ < 0.05).

**Table 1:** Brain regions activated in safe vs. ‘danger+’ conditions (safe > ‘danger+’).

Brain Regions	BA	No. Voxels	Peak t-value	x	y	z
L. Rostral Anterior Cingulate Cortex	10/32/24	1928	5.04	−2	36	−2
Rostral Anterior Cingulate Cortex			4.98	0	38	6
Dorsal Medial Prefrontal Cortex			4.66	−10	62	6
R. Middle Insula	22/13	185	4.57	46	2	−4
Superior Temporal Gyrus			3.98	62	−16	2
Middle Temporal Gyrus			3.94	58	−8	−6
L. Middle Insula	13	37	4.40	−36	2	20
R. Ventral Medial Prefrontal Cortex	11	15	3.97	4	48	−12
L. Anterior Insula	13	24	3.93	−32	10	−10
R. Hippocampus		14	3.69	34	−24	−10
L. Dorsal Medial Prefrontal Cortex		11	3.47	−8	−20	52

All with a P_FDR_ < 0.05 corrected threshold and cluster >10 voxels. MNI coordinates were used. L indicates left; R indicates right.

#### Cue detection trials

**Figure 4:**
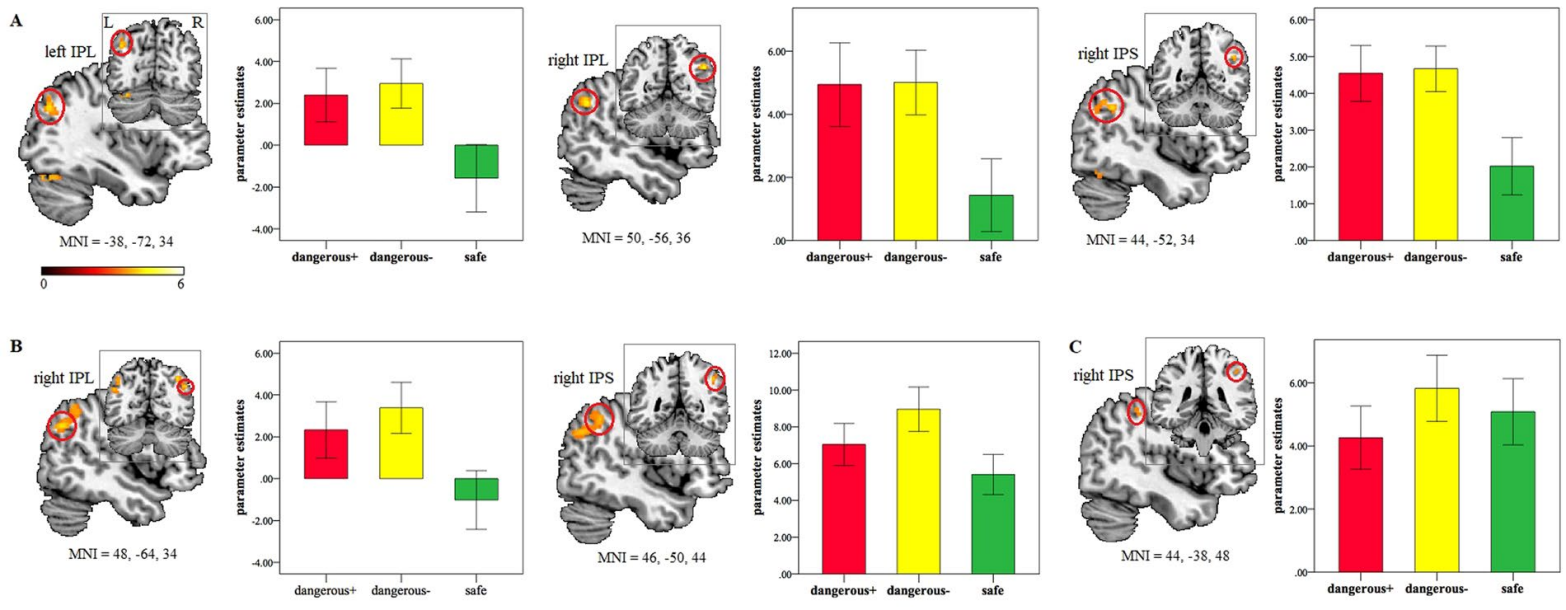
Brain activation in response to (**A**) the D+ relative to safe cues (D+ > safe), (**B**) the D− relative to safe cues (D− > safe) and (**C**) the D− relative to D+ cues (D− > D+). Parameter estimates presented in bar graphs were extracted from a 6-mm sphere centered on the peak coordinates. Statistic maps were displayed with a P < 0.001 uncorrected threshold. IPL: inferior parietal lobule. IPS: intraparietal sulcus. D+: ‘dangerous+’. D−: ‘dangerous−’.

The ROI analysis within the goal directed attentional network during detection of shock probabilty cues showed stronger activity in the bilateral IPL (left: MNI = −38, −72, 34; t = 4.16; P_FWE_ = 0.044; voxels = 36; right: MNI = 50, −56, 36; t = 4.66; P_FWE_ = 0.013; voxels = 85) and the right IPS (MNI = 44, −52, 34; t = 4.15; P_FWE_ = 0.015; voxels = 13) when searching the D+ compared to the safe cues (D+ > safe cue; Fig. 4A). Similar increased activity was also found for the D− relative to the safe cues (D− > safe cue) in the right IPL (MNI = 48, −64, 34; t = 4.23; P_FWE_ = 0.038; voxels = 189) and the right IPS (MNI = 46, −50, 44; t = 4.03; P_FWE_ = 0.020; voxels = 33; Fig. 4B). For the D− vs. D+ comparison (D− > D+ cue), we observed a stronger activation in the right IPS (MNI = 44, −38, 48; t = 3.79; P_FWE_ = 0.037; voxels = 10; Fig. 4C). To further examine this unpredicted finding, we performed an exploratory psychophysiological interaction (PPI) analysis using the PPI toolbox^32^ with the right IPS as a seed region (6-mm sphere centered at MNI = 44, −38, 48). Results showed an increased functional connectivity between the right IPS and the left hippocampus (MNI = −30, −30, −12; t = 4.52; P_FWE_ = 0.023; voxels = 14). We also found increased rACC activation (MNI = 2, 34, 4; t = 4.20; P_FWE_ = 0.027; voxels = 39; Fig. S2) in response to the safe relative to the D+ cues (safe > D+ cue) using a mask from the ‘safe > D+’ contrast (P_FDR_ < 0.05) in RDM trials. No significant effects were observed in the a priori ROIs (P_FWE_ < 0.05) between other comparisons. There were no significant effects in the whole brain analysis (P_FDR_ < 0.05).

## Discussion

The present study investigated how the human brain is organized to search for safe vs. dangerous cues using a novel RDM paradigm combined with threat of electric shocks and whether this process varied as a function of the level of task difficulty. Results on ratings showed that subjects tended to be more confident and less anxious in searching the safe relative to the dangerous cues where they could be shocked for incorrect responses. While we found no evidence for significant effects associated with attentional set in different threat conditions in RDM trials at a behavioral level, subjects responded faster and more accurately in easier than more difficult levels. At the neural level, the vmPFC was recruited both when attention was set to search the safe and D− cues in comparison to the D+ cues. Stronger activation was also found in regions including the inferior frontal gyrus, insula, dmPFC and lingual gyrus for more difficult than easier levels. However, there was no significant interaction between threat conditions and difficulty levels. For cue detection trials, while at a behavioral level subjects were faster and more accurate in detecting the D+ than the D− and safe cues, stronger activity was found in the goal directed attentional networks, including IPL and IPS, for detecting the D+ cues.

Subjects tended to be less confident and more anxious in performing the more threatening relative to the safe searching tasks, which validated the threat manipulation in the present study and coincided with previous findings that the presence of safe cues decreases anxiety to threat^8,9^. Since shock probability cues were presented in a spatiotemporally random way in the current paradigm, it was beneficial for subjects to respond conservatively to increase accuracy, and thus decrease shocks, and this may have contributed to the absence of behavioral differences across threat conditions and the interaction between threat conditions and difficulty levels in RDM trials. For the processing of shock probability cues *per se* in cue detection trials, we found similar patterns for RT and RA with subjects responding faster and more accurately to D+ than to D− and safe cues. These enhanced behavioral responses could be driven by a higher motivational value of D+ cues, as demonstrated by similar preferential processing of threatening stimuli or more valuable stimuli (such as stimuli associated with higher reward)^33,34^.

At the neural level, increased activity was found in the bilateral vmPFC when attention was set to safe relative to the D+ cues in RDM trials, indicating a critical role of the vmPFC in attentional set to safety during visual search. This finding is consistent with the specific role of the vmPFC in learned safety and safety signaling^21–26^. The medial prefrontal areas, especially the vmPFC, receive and integrate inputs from multiple sensory modalities and make them available for higher-level cognitive processes such as emotion regulation, action planning or decision-making^16,35,36^. Thus, high-level information integration in the vmPFC workspace would allow for more efficient vigilance and consequently more elaborate appraisal of potential threat in the environment. In turn, this would facilitate the actions needed to assess safety searching. Note that at whole brain level stronger activity was additionally found in other regions within the ‘cognitive fear’ circuitry including the rACC and hippocampus and in regions including insula and dmPFC, a collective set of hubs implicated in salience processing and fear and anxiety generation^13–16,37–40^, for the safe relative to the D+ threat conditions. These neural circuits may coordinate with each other to enable elaborate assessment of safe cues while monitoring potential threat, thereby generating appropriate levels of anxiety and fear and consequently promoting behavioral flexibility and optimizing survival decisions. Furthermore, the vmPFC, although to a lesser extent, was also recruited in the D− compared to the D+ conditions and overlapped with the vmPFC identified when attention was set to safe cues. Given that subjects could still be shocked in the D− condition, threat vigilance occurring during attentional set to the D− cues could be more comparable with the way distal threat being encoded. However, no evidence was found for interactions between threat conditions and difficulty levels at both behavioral and neural levels, indicating that these attentional set to dangerous or safe cues during visual search were independent of searching load. Together these findings provide the first evidence that the ‘cognitive fear’ circuits, particularly the vmPFC, may be specific substrates underpinning attentional set of searching stimuli that are of survival value in the environment, not only including stimuli signaling safety but also those signaling potential threat.

It is notable that dysfunction of safety processing is also associated with psychiatric disorders^41^, with panic disorder patients showing impaired learning ability in discriminating between safe and dangerous cues and a less effective fear-reduction by safety cues^42^. High trait anxiety individuals also exhibit exaggerated fear generation by safety cues^43^ and post-traumatic stress disorder patients fail to inhibit fear response to safety cues^44^. Furthermore, hyperactivity in the dmPFC and insula regions associated with fear and anxiety generation^37,39,40^, has also been found in individuals with generalized and social anxiety disorders^39,45^. While these two regions might act on generating appropriate anxiety levels to promote behavioral flexibility and optimize survival decisions in the safe condition in healthy populations, as discussed above, the altered activity of these regions could be more closely associated with anxiety symptoms in these psychiatric disorders. Deficient recruitment of the vmPFC has also been reported during fear inhibition in females with generalized anxiety disorder^46^. Thus, these regions could be targets for potential noninvasive therapeutic interventions, such as real-time fMRI neurofeedback training which has been found to be effective at a clinical level^47^.

Consistent with faster and more accurate behavioral responses, stronger activity in the dorsal frontoparietal attention network, including IPL and IPS, was found for the D+ and D− in comparison to the safe cues in cue detection trials. This suggests an enhanced goal-directed attentional processing for more threatening tasks requiring more cognitive resources. These findings coincide with previous studies showing facilitated processing in the attentional network for higher motivational stimuli such as more threatening or rewarding values^30,31,48,49^, which has clear adaptive benefits for survival. Note that the D− cues also induced stronger activity in the right IPS than the D+ cues, which could be driven by an increased functional connectivity of the right IPS with the left hippocampus. This is line with the role of hippocampus either in modulating responses to less imminent threat^1,14,20^ or in orienting visual attention^50,51^. Future studies are necessary to further investigate this question. Furthermore, the rACC was also recruited during processing of the safe compared to the D+ cues. Consistent with its role in attentional set of safety during performance of RDM trials, the enhanced rACC activity may reflect an elaborate evaluation of the utilization of safety, such as a refuge, which is normally associated with protection and opportunity to escape.

There are some limitations in the present study. Firstly, subjects only rated their confidence and pre- and post-anxiety level three times due to the design of the paradigm and thus findings related to these rating effects could be underpowered. Secondly, in the present study there was no physiological measurement of arousal, such as skin conductance response, which could provide an effective index for anxiety/fear levels. Future studies may therefore combine fMRI and physiological recording. Additionally, there is evidence showing that avoidance of an aversive outcome is rewarding and recruits the vmPFC^52^. Thus, the present study can not rule out the possibility that the vmPFC might be involved in successful avoidance of aversive shocks. However, such a potential confound is difficult to overcome as safety is always associated with avoidance of, or the perceived ability to avoid, danger. Finally, the sample size is relatively small, although the power analysis showed an acceptable power level.

Overall, the present study investigated how the human brain encodes safety information by modulating subjects’ attentional set using a novel adaptation of the RDM paradigm. Similar to neural mechanisms involved in processing distal threat, the present study demonstrated that attention set of safety mainly recruited the ventral medial prefrontal regions of the ‘cognitive fear’ circuitry. Thus, encoding of safety signals may share similar neural substrates with processing of distal threat that allows for flexible threat assessment and consequently increases chances of survival for organisms through exploiting their environment. These findings provide new insights into the role of the medial prefrontal regions in the defensive survival system in encoding stimuli with survival significance.

## Methods

### Participants

26 healthy students (13 males, mean age = 22.86 years, SD = 4.03) participated in the present study. All subjects were right-handed and had normal or corrected-to-normal vision. None of them reported a history of, or current neurological or psychiatric symptoms. 4 subjects were excluded due to excessive head motion (1 subject), extremely low response accuracy (RA) to shock probability cues (2 subjects) or technical problems during scanning (1 subject). Thus, 22 subjects were included in the final analysis. A post-hoc power analysis revealed that this sample size achieves a power higher than 80% with an effect size of 0.35 (two-sided α of 0.05), as calculated using the G*Power toolbox^53^. Written informed consent was obtained from all subjects before study inclusion. The study and all procedures were approved by the Columbia University Institutional Review Board and were in accordance with the latest version of the Declaration of Helsinki.

### Stimuli and Procedure

A novel foveal dot-motion paradigm was used in the present study (Fig. 1), which consisted of 3 threat conditions. There were 9 blocks in total with 3 blocks in each threat condition. The threat condition was indicated by a red (‘dangerous+’: D+), yellow (‘dangerous−’: D−), or green square (safe) for 2 s before each block. Subjects were then asked to complete 2 ratings on their confidence and anxiety levels in performing the upcoming blocks using a 1–9 Likert scale within 4 s, followed by a jittered interval of 1–5 s. The block started following the ratings and comprised 22 trials in the same threat condition with a jittered interval of 1–3 s. The 22 trials in each block consisted of 20, 18, or 16 random dots motion (RDM) discrimination trials and corresponding 2, 4, or 6 cue detection trials presented in a pseudorandom order. In the RDM trials, the foveal moving dots stimuli were generated using Psychopy2 software (v1.83)^54^. Each screen consisted of 100 white random dots (10 × 10 pixels) displayed on a grey background with a moving speed of 0.005 frame. Each dot had a lifetime of 25 frames and was randomly assigned a new position after finishing its lifetime. Dots were presented for 2 s with a variety of coherence levels ranging from 9–11%, 14–16%, and 34–36% to the left or right direction corresponding to difficult, moderate and easy levels. The use of different task difficulty levels allowed us to further test if the safety-related effect varied as a function of task difficulty. Dots with these three percentages of coherence moved to the left in half of the trials and to the right in the other half. Subjects were asked to judge the movement direction before they disappeared by pressing either the ‘left’ or ‘right’ response buttons. For incorrect responses, subjects could be punished by one electric shock with an initial probability of 50%, as indicated by a 2-s ‘flash’ symbol feedback. Cue detection trials were designed to manipulate the attentional set in different threat conditions by asking subjects to detect corresponding cues associated with shock probability of RDM trials. The stimuli in the cue detection trial were nearly identical to the RDM trial except that there was a colored shock probability cue presented in a spatiotemporally random way among the moving white dots, and that the feedback display only indicated changes of shock probability. The colored dot was presented for 0.5 s within the 2-s white dots time window. Subjects were informed that they only had to respond to the shock probability cue when it appeared and were instructed to respond as fast as possible before it disappeared. In the D+ condition (red dot), while correct responses did not change the shock probability each incorrect response increased it by 10%. In the D− condition (yellow dot), while each correct response decreased the shock probability by 10%, incorrect responses did not change it. Shock probability changes were indicated by a 2-s outcome feedback. The feedback was a ‘flash’ symbol with specific shock probability changes (e.g., ‘+10%’ or ‘−10%’) appearing above the symbol and the current shock probability below it. In the safe condition (green dot), there was no shock and incorrect responses were given a 2-s ‘cross’ feedback. Only responses with reaction times (RTs) shorter than 650 ms were categorized as correct. These colored dots were carefully counterbalanced in luminance and size. To further show that there was no perceptual bias for the different colored dots, we also conducted a control experiment with an independent sample (N = 18) using a similar dot-motion paradigm, but without administering shock. This showed that there was no significant effect across the different colored dots on either response RTs (ps > 0.196) or RA (ps > 0.210). To maintain maximal continuous attention set for searching of shock probability cues, subjects were clearly informed that the colored dot could appear at any time point during the 2-s display of white dots on the screen and that only a fast enough correct response would be regarded as a ‘real’ correct response. Thus to minimize the shock probability, subjects had to maintain their attentional set to shock probability cues even during performing the RDM trial. Following the outcome feedback, subjects were asked to rate their anxiety level while performing the task.

### Image Acquisition and Data Analysis

Images were collected using a 3 T, GE Discovery MR750 scanner (General Electric Medical System, Milwaukee, WI, USA). For the fMRI scan, a time series of volumes was acquired using a T2*-weighted EPI pulse sequence (repetition time, 2000 ms; echo time, 25 ms; slices, 45; thickness, 3 mm; field of view, 192 × 192 mm; resolution, 64 × 64; flip angle, 77°). High-resolution whole-brain volume T1*-weighted images (1 mm isotropic resolution) were acquired obliquely with a three-dimensional spoiled gradient echo pulse sequence before the fMRI scan.

Brain images were processed using the SPM8 software package (Wellcome Department of Cognitive Neurology, London, UK, http://www.fil.ion.ucl.ac. uk/spm/spm8)^55^. The first five images were excluded to achieve magnet-steady images and the remaining functional images were realigned to correct for head motion based on a six-parameter rigid body algorithm. After co-registering the mean functional image and the T1 image, the T1 image was segmented to determine the parameters for normalizing the functional images to Montreal Neurological Institute (MNI) space. Next normalized images were spatially smoothed with an 8 mm full-width at half maximum of Gaussian kernel.

The first-level design matrix included 19 regressors (threat condition cue, confidence rating, pre-anxiety rating, three threat conditions at each difficulty level, three colored threat dots, outcome feedback of RDM trials, outcome feedback of cue detection trials, delivered shocks, post-anxiety rating) and the 6 head-motion parameters convolved with the canonical hemodynamic response function. On the first level for RDM trials, contrast images for each condition (D+/D−/safe condition at the difficult/moderate/easy level separately) were created for each subject. Based on these specific contrasts, an ANOVA model implemented in a flexible factorial design was used on the second level for RDM trials to examine the difference of attentional set between D+, D− and safe conditions, particularly the neural effect associated with attentional set to safety in contrast to the D+ and D− conditions. This design also allowed us to test the interaction between threat condition and difficulty. On the first level for cue detection trials, contrast images for shock probability cues in each threat condition (D+/D−/safe condition) were created for each subject. Then a one-way ANOVA within subject design on the second level was used to test the difference between shock probability cues in the D+, D− and safe conditions. For the whole brain analysis, a significance threshold of P < 0.05 false discovery rate (FDR) correction was used with a minimum cluster size of 10 contiguous voxels.

To examine the safety-related effect of attentional set in RDM trials in a more sensitive way, we further performed a hypothesis-driven region of interest (ROI) analysis in the vmPFC, which has been shown as a core region of safety signaling^21–26^. Furthermore, for cue detection trials, we additionally included ROIs involved in goal-directed attentional processing, namely the intraparietal sulcus (IPS), the inferior parietal lobule (IPL) and frontal eye field, which are core hubs of the frontoparietal attention network^27–29^. The vmPFC was derived from the Automated Anatomic Labeling atlas^56^. The SPL, IPL and IPS were derived from probabilistic maps implemented in Anatomy toolbox 2.1^57^. Within these a priori ROIs, a small volume correction with a threshold of p < 0.05 family-wise error (FWE) corrected at peak level was set for multiple comparisons.

## Electronic supplementary material

Supplementary Information

## Data Availability

The datasets generated during and/or analyzed during the current study are available from the corresponding author on reasonable request.
