# Supplementary material for: Attentional set to safety recruits the ventral medial prefrontal cortex
Source: Sci Rep. 2018 Oct 18;8:15395. doi: 10.1038/s41598-018-33953-3 (PMC6193957; doi:10.1038/s41598-018-33953-3)
Supplement: Supplementary file 1 — Supplementary Information [file 41598_2018_33953_MOESM1_ESM.docx]

**Supplementary Information**

**Attentional set to safety recruits the** **ventral medial prefrontal cortex**

Shuxia Yao^1^, Song Qi^2,3^, Keith M. Kendrick^1^*, Dean Mobbs^2,3^*

^1^The Clinical Hospital of Chengdu Brain Science Institute, MOE Key Laboratory for NeuroInformation, University of Electronic Science and Technology of China, Chengdu, Sichuan 611731, China

^2^California Institute of Technology, Pasadena, California 91125

^3^Columbia University in the City of New York, New York, NY 10027

**
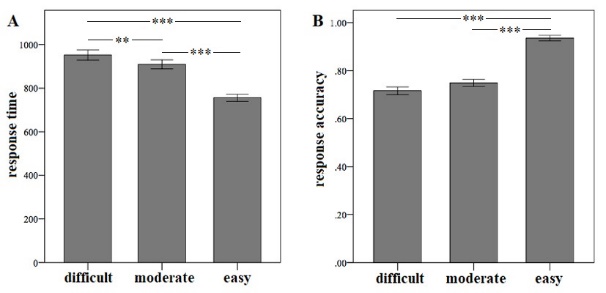
Figure S1. Response time (A) and accuracy (B) to different difficulty levels in random dots motion discrimination trials.** *P < 0.05; **P < 0.01; *** P < 0.001

**
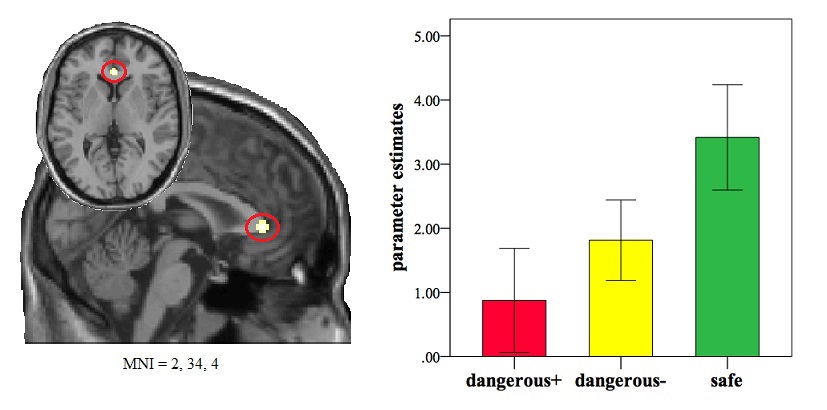
Figure S2. Increased rostral anterior cingulate cortex in response to the safe relative to ‘dangerous +’ threat cues (safe > ‘dangerous +’) in cue detection trials.** Parameter estimates presented in bar graphs were extracted from a 6-mm sphere centered on the peak coordinates. Statistic maps were displayed with a P < 0.001 uncorrected threshold.

**Table S1. Brain regions related to task difficulty.**

| Brain Region | BA | No. Voxels | Peak t-value | x | y | z |
| --- | --- | --- | --- | --- | --- | --- |
| **Difficult > Easy** |  |  |  |  |  |  |
| R. Inferior Frontal Gyrus | 13/47/45 | 567 | 7.44 | 34 | 26 | 4 |
| Anterior Insula |  |  | 6.73 | 42 | 20 | -2 |
| Inferior Frontal Gyrus |  |  | 6.25 | 36 | 24 | -8 |
| R. Inferior Frontal Gyrus | 6/9 | 1264 | 7.38 | 52 | 10 | 34 |
| Middle Frontal Gyrus |  |  | 6.90 | 36 | -4 | 48 |
| L. Lingual Gyrus | 18/19/7 | 4150 | 7.38 | -14 | -92 | -12 |
| Inferior Temporal Gyrus |  |  | 7.35 | -48 | -70 | -4 |
| Middle Temporal Gyrus |  |  | 7.34 | -28 | -92 | -6 |
| R. Lingual Gyrus | 18/19/17 | 1263 | 6.90 | 14 | -92 | -8 |
| Middle Temporal Gyrus |  |  | 6.33 | 32 | -68 | 22 |
| Inferior Occipital Gyrus |  |  | 6.24 | 38 | -84 | -6 |
| R. Dorsal Medial Frontal Gyrus | 6/32/8 | 983 | 7.07 | 4 | 10 | 52 |
| Middle Cingulate Gyrus |  |  | 6.59 | 6 | 16 | 44 |
| Dorsal Medial Frontal Gyrus |  |  | 6.52 | 4 | 22 | 50 |
| L. Inferior Frontal Gyrus | 6/9 | 668 | 6.76 | -46 | 2 | 32 |
| Middle Frontal Gyrus |  |  | 6.35 | -30 | -8 | 48 |
| Precentral Gyrus |  |  | 5.65 | -44 | -4 | 50 |
| R. Inferior Parietal Lobule | 7/40 | 889 | 6.64 | 34 | -48 | 50 |
| Superior Parietal Lobule |  |  | 6.43 | 30 | -56 | 46 |
| Inferior Parietal Lobule |  |  | 6.02 | 40 | -40 | 48 |
| L. Anterior Insula | 13/47/45 | 319 | 6.59 | -32 | 22 | 6 |
| Inferior Frontal Gyrus |  |  | 6.12 | -30 | 22 | -6 |
| Anterior Insula |  |  | 5.73 | -38 | 16 | 0 |
| R. Caudate |  | 364 | 6.15 | 10 | 6 | 4 |
| Thalamus |  |  | 5.91 | 6 | -20 | 8 |
| R. Lingual Gyrus | 30/18 | 145 | 5.85 | 22 | -60 | 2 |
| Cuneus |  |  | 5.47 | 16 | -66 | 6 |
| L. Lingual Gyrus | 30/19 | 44 | 5.47 | -24 | -64 | 0 |
| Cuneus |  |  | 5.00 | -18 | -68 | 4 |
| L. Extra-Nuclear |  | 38 | 5.35 | -8 | 2 | 6 |
| Thalamus |  |  | 5.06 | -2 | -6 | 6 |
| **Easy > Difficult** |  |  |  |  |  |  |
| None |  |  |  |  |  |  |
| **Difficult > Moderate** |  |  |  |  |  |  |
| L. Caudate |  | 93 | 5.86 | -8 | 2 | 8 |
| R. Caudate |  | 59 | 6.11 | 8 | 4 | 6 |
| L. Lingual Gyrus |  | 15 | 5.16 | -20 | -96 | -12 |
| R. Lingual Gyrus | 17 | 22 | 5.43 | 14 | -94 | -8 |
| R. Thalamus |  | 41 | 5.26 | 8 | -18 | 12 |
| L. Middle Occipital Gyrus | 37/19 | 26 | 5.21 | -50 | -76 | 4 |
| R. Precentral Gyrus | 4 | 16 | 5.21 | 40 | -18 | 48 |
| R. Middle Temporal Gyrus | 39/19 | 24 | 5.21 | 52 | -70 | 10 |
| R. Dorsal Medial Frontal Gyrus | 9 | 26 | 5.17 | 2 | 52 | 26 |
| L. Inferior Temporal Gyrus | 37 | 15 | 5.11 | -48 | -70 | -6 |
| **Moderate > Difficult** |  |  |  |  |  |  |
| None |  |  |  |  |  |  |
| **Moderate > Easy** |  |  |  |  |  |  |
| R. Middle Frontal Gyrus | 9 | 72 | 5.39 | 52 | 12 | 34 |
| Inferior Frontal Gyrus |  |  | 5.00 | 56 | 20 | 28 |
| R. Inferior Frontal Gyrus | 7/40 | 36 | 5.27 | 34 | -48 | 50 |
| L. Precuneus | 7 | 65 | 5.15 | -24 | -60 | 40 |
| Superior Parietal Lobule |  |  | 5.00 | -26 | -64 | 50 |
| L. Lingual Gyrus | 17 | 21 | 5.14 | -14 | -92 | -12 |
| R. Dorsal Medial Frontal Gyrus | 6 | 14 | 5.06 | 4 | 12 | 52 |
| L. Middle Occipital Gyrus | 18 | 14 | 4.84 | -28 | -92 | -4 |
| **Easy > Moderate** |  |  |  |  |  |  |
| None |  |  |  |  |  |  |

All with a P_FWE_ < 0.05 correction threshold and cluster > 10 voxels at whole brain level. MNI coordinates were used. L indicates left; R indicates right.
